# Beyond Single-Cell Analysis of Metallodrugs by ICP-MS: Targeting Cellular Substructures

**DOI:** 10.3390/ijms22179468

**Published:** 2021-08-31

**Authors:** Audrey Galé, Lukas Hofmann, Nicola Lüdi, Martin Nils Hungerbühler, Christoph Kempf, Johannes Thomas Heverhagen, Hendrik von Tengg-Kobligk, Peter Broekmann, Nico Ruprecht

**Affiliations:** 1 Department of Diagnostic, Interventional and Pediatric Radiology, Bern University Hospital, University of Bern, 3010 Bern, Switzerland; audrey.gale@students.unibe.ch (A.G.); martin.hungerbuehler@dbmr.unibe.ch (M.N.H.); christoph.kempf@dbmr.unibe.ch (C.K.); johannes.heverhagen@insel.ch (J.T.H.); hendrik.vontengg@dbmr.unibe.ch (H.v.T.-K.); 2 Department for BioMedical Research DBMR, University of Bern, 3008 Bern, Switzerland; 3 Institute of Nanotechnology and Advanced Materials & Department of Chemistry, Faculty of Exact Sciences, Bar-Ilan University, Ramat-Gan 5290002, Israel; hofmmal@biu.ac.il; 4 Department of Chemistry, Biochemistry and Pharmaceutical Sciences (DCBP), University of Bern, 3012 Bern, Switzerland; nicola.luedi@unibe.ch

**Keywords:** NSCLC, cisplatin, chemotherapy, single cell ICP-MS, cisplatin resistance, nuclear uptake

## Abstract

Platinum compounds such as cisplatin (cisPt) embody the backbone of combination chemotherapy protocols against advanced lung cancer. However, their efficacy is primarily limited by inherent or acquired platinum resistance, the origin of which has not been fully elucidated yet, although of paramount interest. Using single cell inductively coupled plasma mass spectrometry (SC-ICP-MS), this study quantifies cisPt in single cancer cells and for the first time in isolated nuclei. A comparison of cisPt uptake was performed between a wild type (wt) cancer cell line and related resistant sublines. In both, resistant cells, wt cells, and their nuclei, cisPt uptake was measured at different incubation times. A lower amount of cisPt was found in resistant cell lines and their nuclei compared to wt cells. Moreover, the abundance of internalized cisPt decreased with increasing resistance. Interestingly, concentrations of cisPt found within the nuclei were higher than compared to cellular concentrations. Here, we show, that SC-ICP-MS allows precise and accurate quantification of metallodrugs in both single cells and cell organelles such as nuclei. These findings pave the way for future applications investigating the potency and efficacy of novel metallodrugs developed for cancer treatment.

## 1. Introduction

Since the serendipitous discovery of cisplatin (cisPt) and its anticancer properties by Rosenberg in 1965 and its subsequent approval as therapeutic in 1978, numerous platinum compounds have been synthesized and tested. However, only two more platinum-based drugs were approved worldwide since then. These two drugs were carboplatin and oxaliplatin [1]. The group of Barnett Rosenberg discovered the robust antiproliferative effects of cisPt in the late 1960s. This antiproliferative effect was explained by a complex formation between Pt(II) and nitrogen atoms of nucleotide bases (platinum-DNA adducts), that ultimately induces kinks and partial unwinding of the DNA helix [2]. This conformational change in the DNA structure halts the cell cycle and initiates programmed cell death [3,4]. Passive diffusion was considered as the sole mechanism for cisPt entering the cell [5,6]. However, in recent years alternative uptake pathways such as the Copper transporter 1 (CTR1) was identified as an important transmembrane protein involved in cisPt uptake [7,8]. Furthermore, copper-extruding P-type ATPases were found to reduce the cytoplasmic or cellular cisPt concentration [9,10]. Recently, Planells-Cases et al. [11] described that loss of LRRC8A and LRRC8D subunits of the volume-regulated anion channel (VRAC) effects cisPt uptake. Thus, the mechanisms involved in acquired resistance to cisPt likely comprise a variety of transition metal transporters and other yet unknown proteins present in mammalian cells.

Inherent or acquired platinum resistance is a major limitation to improve long-term outcomes in cancer therapy. Recent discoveries described several novel resistance mechanisms [12,13,14], which are divided into two groups. The first group demonstrates an insufficient uptake of platinum into cancer cells thus resulting in a lower cisPt concentration in the cytoplasm and a reduced amount of platinum-DNA adducts. The second group of resistance mechanisms relies on a failure of the apoptosis initiation process due to erroneous platinum-DNA adduct formation. To overcome cisPt resistance in anti-cancer therapy numerous approaches have been pursued [14]. Unfortunately, none of these approaches was implemented into clinics so far [15]. Therefore, the newly proposed VRAC as an importing channel for cisPt and carboplatin provides a novel promising target circumventing platinum-drug resistance in cancer patients [16].

With nearly 1.5 million new cases diagnosed worldwide each year, lung cancer is the most frequent diagnosed cancer in humans, the most common, aggressive, and also deadliest type of cancer worldwide [15,17]. There are two main types of this disease, non-small cell lung cancer (NSCLC) and small cell lung cancer (SCLC). NSCLC accounts for 85% of all cases and is the most common type of lung cancer [15]. The current five year survival rates are 15–20%. Among patients with NSCLC, these rates reach 90% for stage 1 but drop below 10% for stage 4. Among patients with SCLC, the rates are about 30% for limited disease and below 10% for extensive disease [18]. Chemoimmunotherapy is currently being explored to improve first-line chemotherapy doublets by adding, e.g., immune checkpoint inhibitors [19].

To date, different cisPt resistant cell lines of various cancer types have been described [20,21,22]. Recently, cisPt resistant A240286S (A24) NSCLC sublines have been established. Clinically relevant resistance grades showed resistance levels above 4.0 µM cisPt. This resistance persisted even after more than one year in absence of cisPt. However, a correlation between cisPt resistance and expression level of VRAC transporter in the A24 cell line was not confirmed [23]. Thus, loss of the VRAC transporter is obviously not the primary origin of the cisPt resistance in A24 cells. Hence, these recent findings are conflicting with previously published studies for other cell lines [11,24,25,26]. These fundamental controversies demonstrate that acquisition of transition metal drug resistance remains enigmatic to us, albeit of paramount interest. Therefore, only a comprehensive understanding of the acquisition of resistance allows for a targeted precision treatment of patients suffering from deadly lung cancer metastasis.

Here, we show exemplary that implementation and development of single cell inductively coupled plasma mass spectrometry (SC-ICP-MS) have paved the way for a novel area of anti-cancer research. This analysis technique enables immediate detection and quantification of metal-based compounds on the level of single cells. Because each cell is unique, is in its own growth stage, and the cell population has a distribution of different cell volumes, the use of SC-ICP-MS is particularly useful in the context of these experiments. In this study, SC-ICP-MS has proven to be a robust alternative to conventional ICP-MS for the quantification of intracellular platinum. By applying this method, the cells dissolved in water and PBS remain intact, thus preventing the loss of intracellular metal due to membrane permeabilization [27]. In this present study, the accumulation of cisPt is quantified by means of SC-ICP-MS in sensitive, resistant, and for the first time in the nuclei of A24 cells.

## 2. Results

### 2.1. Determination of Intracellular cisPt in Wild Type (wt) and Resitant Cells Using SC-ICP-MS

**Figure 1:**
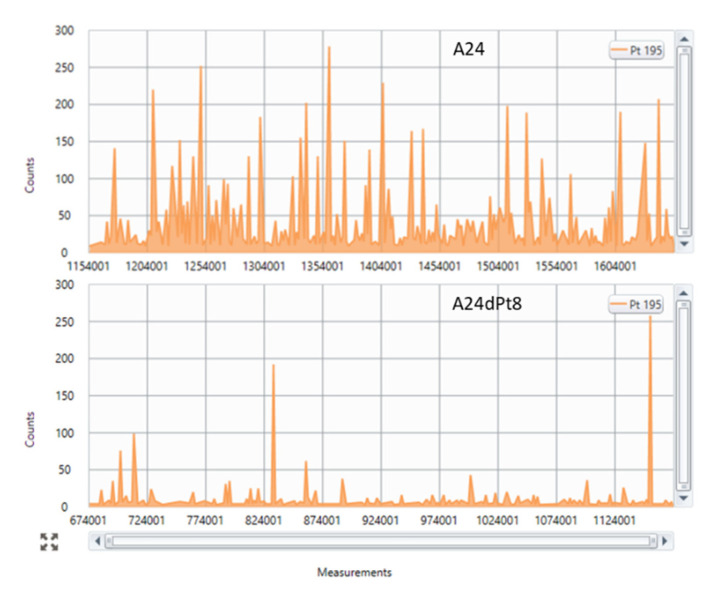
Single cell inductively coupled plasma mass spectrometry (SC-ICP-MS) representative sections of (Pt 195) counts versus time traces in A24 cells and A24dPt8 cells. This figure shows real-time signal processing by Syngistix. It becomes apparent that less Pt is measured in resistant cells (bottom panel) than compared to wild type (wt) cells (top panel), independent of the measured time point (*x*-axis). Each “intensity peak” represents accumulated Pt 195 counts for a defined time interval (dwell time) of 50 μs. Each time interval correspond to one “single measurement” (*x*-axis). Assumption: each peak (measurement) corresponds to one individual entity analyzed (cell, cells aggregate).

The concentration of cisPt was determined in sensitive A24 wt cells and cisPt resistant A24dPt8 (previously described as (D-) Pt8.0A24 [23]) single cells with cisPt exposure of 30 µM for 0, 2, 4, 8, and 16 h. After more than two years in culture lacking cisPt, the IC_50_ value of resistant A24 cells was re-evaluated and still revealed an IC_50_ of 12.74 µM. Figure 1 shows a representative section of the counts vs. measurements in sensitive A24 wt cells and resistant A24dPt8 treated with 30 µM cisPt for 16 h, respectively.

**Figure 2:**
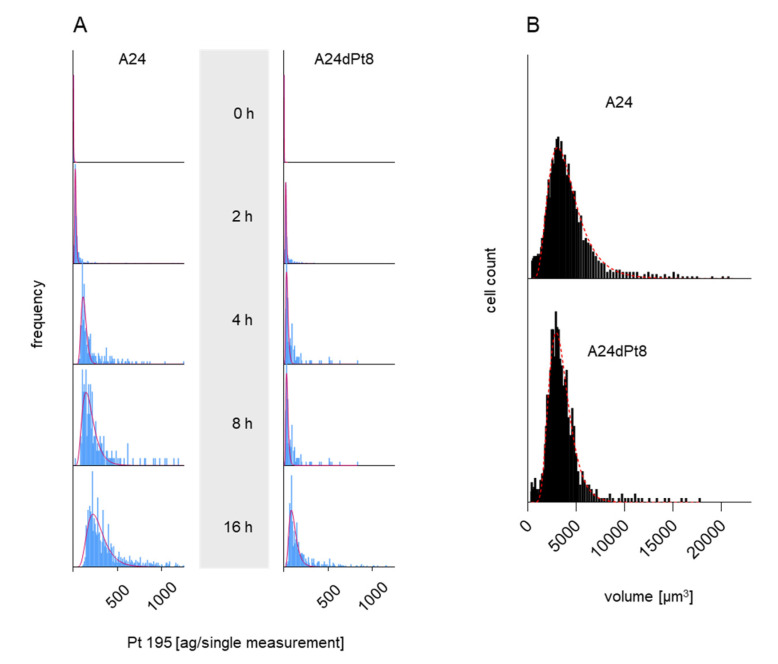
Frequency plot (integrated/fitted histogram; Syngistix-program *PerkinElmer*) of A24 and A24dPt8 cells (**A**) incubated with 30 µM cisPt for 0, 2, 4, 8, and 16 h. (**B**) Volume distribution vs. cell count (frequency). The red curves in (**A**) and (**B**) show the lognormal fit of the frequency.

Frequency distribution plots showing cisPt masses versus number of cell events (frequency) were obtained for 0, 2, 4, 8, and 16 h in presence of 30 µM cisPt as shown in Figure 2A. Strikingly, longer incubation time, led to an increased amount of cisPt per A24 cells and in A24dPt8 cells. Additionally, varying cisPt amounts per cell were observed within A24 sublines, indicating that cisPt bioavailability of sensitive and resistant A24 cells varies within the cell population. The cellular concentration was calculated with the volume of A24 or A24dPt8 cells for each cell population (Figure 2B). Volumes up to 14,000 µm^3^ were measured with a mean of 3800 µm^3^ for A24 cells and 3200 µm^3^ for A24dPt8 cells.

### 2.2. Intracellular cisPt Concentration in A24 and A24dPt8 Cells

Prior to the SC-ICP-MS measurement, the volume of the targeted cells had to be determined as basis for the proper calculation of the (mean) cellular cisPt concentration. SC-ICP-MS measurements, carried out for A24 and A24Pt8 cells, revealed an increasing trend of the cisPt concentration with exposure (incubation) time in case of A24 cells as well as A24dPt8. However, increase in intracellular Pt in A24dPt8 was much less prominent compared to the A24 cells.

**Figure 3:**
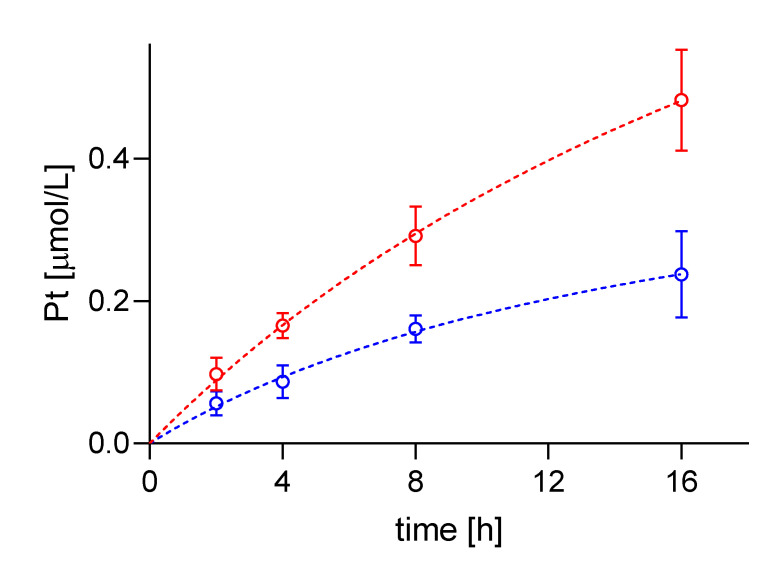
Timeline of cisPt uptake by --●-- A24 and --●-- A24dPt8 cells after incubation with 30 µM cisPt.

Average cisPt concentration determined by SC-ICP-MS in A24 cells was 0.1 ± 0.02 µM cisPt per cell after an incubation time of 2 h. Measurements at 4, 8, and 16 h of incubation, resulted in 0.17 ± 0.02, 0.29 ± 0.04, and 0.48 ± 0.07 µM cisPt/cell, respectively. In contrast, determination of cellular cisPt concentration in A24dPt8 revealed an average concentration of 0.06 ± 0.02 µM cisPt per cell after 2 h. Measurement at 4 h showed an average of 0.09 ± 0.02 µM cisPt, at 8 h 0.16 ± 0.02 µM cisPt, and at 16 h 0.24 ± 0.06 µM cisPt/cell (Figure 3).

The observed ratios between the A24 and A24dPt8 cells were approximately two-fold higher in the wt cells compared to the resistant cells for each observed time point.

Comparison of cisPt concentration in A24 cells and resistant cells showed that the mean cellular cisPt concentrations per cell were significantly different. Statistical analysis was performed with a multiple unpaired t-test. Statistical significance was determined by the Holm–Sidak method with alpha = 0.05 (Holm 1979). The *p* values obtained were < 0.02 at 2 h incubation time with cisPt and < 0.001 for 4, 8, and 16 h, respectively.

### 2.3. Difference in cisPt Uptake between wt and Resistant Cells

**Figure 4:**
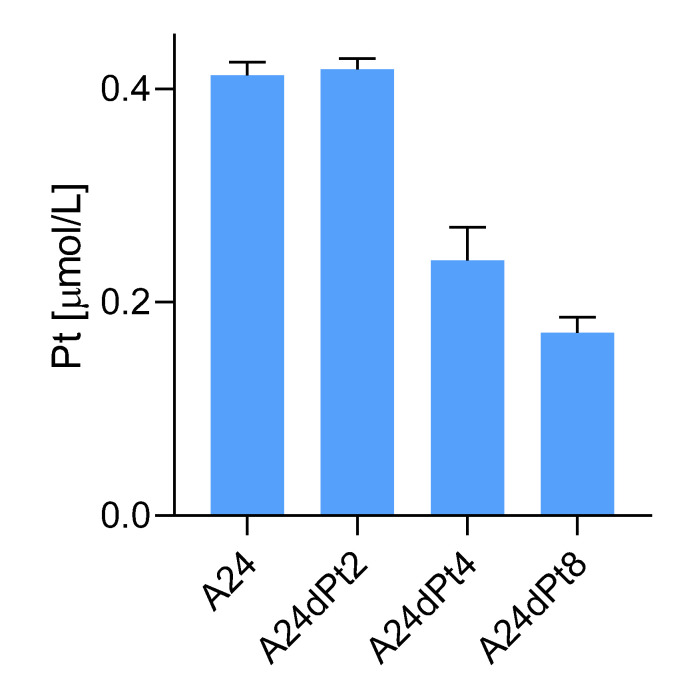
Cellular cisPt concentration in A24, A24dPt2, A24dPt4, and A24dPt8 cells after 16 h incubation with 30 µM cisPt.

Altered cisPt uptake among different sublines with graduated cisPt resistance was assessed in sublines A24dPt2 and A24dPt4 (previously described as (D-)Pt2.0A24 and (D-)Pt4.0A24 [23]), A24dPt8 and A24 cells incubated for 16 h with 30 µM cisPt. The previously measured volume of the cells allowed for determination of the cellular cisPt concentration by SC-ICP-MS. No difference was detected in the uptake of cisPt between the A24 cells and the A24dPt2 cells with approximately 0.42 µM cisPt per cell (*p* > 0.05). However, significantly lower cisPt uptake was observed for the A24dPt4 and A24dPt8 sublines with 0.24 ± 0.03 (*p* < 0.01), and 0.17 ± 0.01 µM (*p* < 0.01) cisPt, respectively (Figure 4).

### 2.4. Nuclear cisPt Concentration in A24 and A24dPt8 Cells

**Figure 5:**
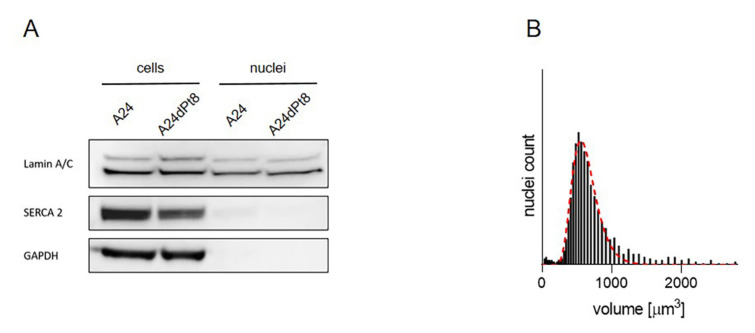
Isolation of intact nuclei. (**A**) The purity of the nuclear fraction was assessed using Lamin A/C (nuclear marker), SERCA2 ATPase (ER marker) and GAPDH (cytoplasmic marker) by Western blot. Column 1 and 2 show the cell extracts of A24 and A24dPt8, column 3 and 4 show the A24 isolated nuclei and the A24dPt8 isolated nuclei. (**B**) Representative volume distribution of isolated nuclei from A24 cells. The red curve shows the lognormal fit of the frequency.

As previously described [2], the cytotoxic action of cisPt on NSCLC is induced by binding of cisPt to DNA. Nuclei were isolated from cells that were treated with 30 µM cisPt. Assessment of the purity of the nuclear extract was performed by Western blot verifying the absence of endoplasmic reticulum and cytosolic contaminants (Figure 5A). A nuclear marker (Lamin A/C, 63 and 74 kDa), an endoplasmic reticulum marker (SERCA 2 ATPase, 115 kDa), and a cytosolic marker (GAPDH, 40.2 kDa) were applied. Whole cell extracts, washed in PBS and re-suspended in RIPA buffer were used as positive controls (Figure 5A). Nuclear concentrations of cisPt were then calculated according to nuclei volumes (Figure 5B). Volumes up to 1800 µm^3^ were observed with a mean of 600 µm^3^ per nucleus in both cell sublines A24 wt and A24dPt8, respectively.

**Figure 6:**
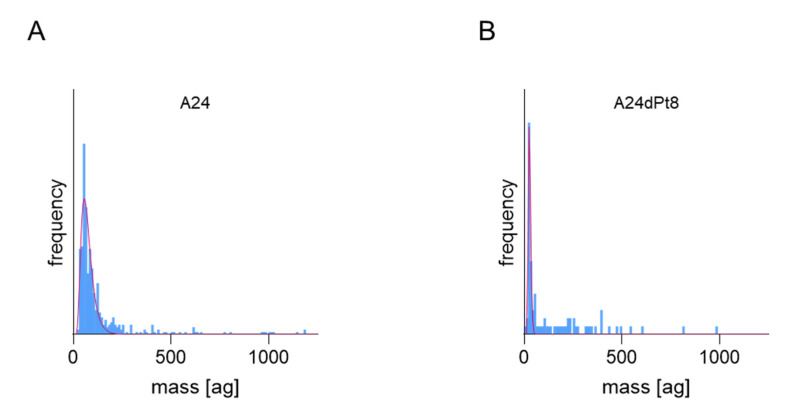
Frequency plots of isolated nuclei from (**A**) A24 cells and (**B**) A24dPt8 cells incubated with 30 µM cisPt for 16 h. The red curve shows the lognormal fit of the frequency.

SC-ICP-MS was used to determine the concentration of cisPt in nuclei of A24 and A24dPt8 cells. For this purpose, A24 and A24dPt8 cells were exposed to 30 µM cisPt for 8 and 16 h. Subsequently, the cell nuclei were isolated and the amount of platinum per nucleus was measured. Similar to the cisPt concentration per cell, A24 and A24dPt8 nuclei showed varying amounts of cisPt within cell populations. Frequency histograms for 16 h incubation with cisPt obtained by SC-ICP-MS analysis are shown in Figure 6. Varying amounts of cisPt per nuclei were measured within both A24 and A24dPt8.

**Figure 7:**
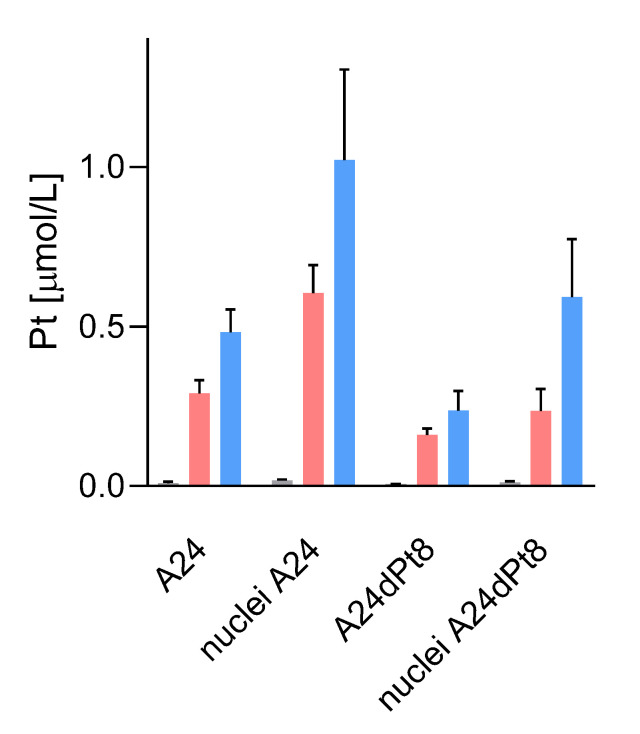
Nuclear and cellular cisPt concentration after incubation of A24 and A24dPt8 cells for ▐ 0, ▐ 8, and ▐ 16 h with 30 µM cisPt.

Average concentration of cisPt in nuclei of sensitive and resistant cells were determined, using measured nuclear volumes. The average cisPt concentration detected by SC-ICP-MS for 8 h in A24 cells was 0.6 ± 0.08 µM cisPt per nucleus, and 1.02 ± 0.27 µM cisPt per nucleus after 16 h of incubation. In contrast, determination of nuclear cisPt concentration in resistant A24dPt8 cells revealed an average concentration of 0.23 ± 0.06 µM cisPt per nucleus at 8 h. At 16 h, an average of 0.59 ± 0.17 µM cisPt per nucleus was detected. CisPt concentrations measured in nuclei or cells within the same cell line are significantly higher in nuclei than in cells. From SC-ICP-MS measurements, an approximate two-fold higher concentration of cisPt was found in nuclei compared to A24 cells at 8 and 16 h, respectively (Figure 7).

## 3. Discussion

In the present study, cisPt accumulation in cells and nuclei of sensitive and resistant A24 sublines was analyzed by SC-ICP-MS. To date, platinum compounds are the gold standard of combined chemotherapy protocols for advanced lung cancer. ICP-MS is an element-specific analytical method that allows tracking of metallodrugs in biological samples and has become a routine method to determine drug accumulation and/or distribution in tissues or cells. However, cells can have significant heterogeneity, resulting in variable metal uptake. When performing quantitative analysis of metallodrugs by means of ICP-MS following digestion of targeted cells, this information is typically lost. However, SC-ICP-MS overcomes these conceptual shortcomings of standard analysis approaches, since it is capable to detect metal (e.g., Pt) contents of individual cells and therefore permits tracking and quantification of metallodrugs in biological samples [27]. The use of SC-ICP-MS allowed both, to determine the cisPt content of single cells and to quantify the cisPt present in individual extracted nuclei. Analysis of the cisPt concentration in both, cells and isolated nuclei, volume distribution of cell population and nuclei was considered.

Uptake of cisPt was determined in platinum-sensitive lung adenocarcinoma wt cells from an adrenal metastasis and the corresponding cisPt-resistant sublines. In a first series of experiments, SC-ICP-MS was used to measure the uptake of cisPt in A24 and A24dPt8 cells over time (Figure 2 and Figure 3). A high mass dispersion was observed between A24 cells and A24dPt8 cells following a lognormal distribution. The volume was found to vary within cell populations and to follow a lognormal distribution as well (Figure 2B). It should also be kept in mind that this measurement method cannot exclude the possibility of cell aggregation, even though cells were passed through a 25-gauge needle prior to measurement. Since both cisPt uptake and cell volumes follow a lognormal distribution, the respective mean values were used to calculate intracellular cisPt concentrations.

Over time, an increase of the cisPt concentration was observed for both A24 and A24dPt8 cells. However, the uptake is significantly lower in the A24dPt8 cell line than compared to the wt-A24 cell line. Approximately twice the concentration of cisPt was measured in A24 wt cells as compared to resistant A24dPt8 cells at different time points. This difference in cisPt uptake is consistent with several studies comparing cisPt accumulation in resistant and sensitive cell lines [28,29,30,31,32]. The difference in concentration of cisPt in resistant cells compared to A24 wt cells can be explained by less cisPt uptake, excretion, or both. In different cell types, under-expression of membrane transporters or overexpression of drug efflux pumps contributes to less Pt accumulation within the cell [33,34]. After incubation with 30 µM cisPt for 0, 2, 4, 8 and 16 h, the resulting curves for A24 and A24dPt8 show a pattern of saturation for cisPt in the resistant and sensitive cell sublines.

Uptake of cisPt was measured after 16 h of incubation with 30 µM cisPt in A24, A24dPt8, and two additional resistant cell lines A24dPt2 and A24dPt4 (Figure 4). The intracellular concentration of cisPt decreased with increased resistance. A24dPt8 showed a concentration of 0.17 µM, while A24 showed concentrations above 0.4 µM. Intermediate resistant cells A24dPt4 showed intracellular concentrations of cisPt (0.24 µM). A24dPt2 cells exhibited intracellular concentrations of cisPt similar to A24. Hence, uptake of cisPt correlates with resistance of the cell subline cultured in increasing concentrations of cisPt for a prolonged time. Because resistance due to cisPt induction of cells increases, the uptake of cisPt decreases. These findings suggest that cisPt resistance is partially due to reduced cisPt import by the cells. This reduction could be explained by an altered transport mechanism of cisPt into the cells or by an additional excretion mechanism. However, lower uptake of cisPt is not sufficient to explain the development of resistance to cisPt. This is because a significant concentration of cisPt was still measured in resistant cells. Thus, epigenetic modifications, e.g., DNA methylation [33,35], structural changes in chromatin [33,36,37] or histone modifications [33,38] may confer resistance to cisPt. Additional mechanisms of resistance to cisPt have been reported, such as transcription factors [33,39], gene regulation by microRNA [33,40], or expression of proteins involved in susceptibility or against DNA damage [33].

According to previous studies, cisPt targets the nucleus by binding to nucleotide base nitrogen residues [2]. In ovarian cancer cells (A2780), concentrated levels of Pt were found in the nucleus with hotspots in the nucleolus, which may suggest a role for cisPt in modulating protein production through interactions with RNA and ribosome synthesis [33]. To our knowledge, cell organelles, namely cell nuclei, were investigated for the first time by SC-ICP-MS. Purified nuclei from A24 or A24dPt8 cells treated initially with 30 µM cisPt were analyzed by Western blot verifying the purity (Figure 5). CisPt uptake in cell nuclei was determined by SC-ICP-MS (Figure 7). Comparable to the whole cell analysis, the dispersion was more pronounced in A24 wt cell nuclei compared to A24dPt8 cell nuclei. The cell nuclei size was heterogeneous; hence, the average volume was used to calculate the concentration of cisPt. A significantly higher concentration of cisPt was determined in the A24 wt cell nuclei compared to A24dPt8 cell nuclei. The concentration of cisPt was approximately two-fold higher in the A24 wt nuclei than compared to resistant nuclei. However, the cisPt present in the resistant cells is imported into the nucleus at approximately the same ratio as in A24 cells. It was found that the concentration of cisPt within the nucleus is approximately two-fold higher compared with the cell concentration in A24 cells and in A24dPt8 cells. This observation supports the theory of binding of cisPt to DNA, resulting in a higher concentration of cisPt in cell nuclei compared to the extranuclear cell space. The increase in Pt concentration within the nucleus becomes even more prominent if the mass balance, that can be calculated from the concentration and volumes of the cells and nucleus, respectively, is considered. Furthermore, we concluded that the uptake mechanism of cisPt into the nucleus of resistant cells is not different from that of sensitive cells, and passive diffusion occurs most probably through the nuclear pore [41]. The amount of cisPt detected in the nuclei of resistant cells after 16 h of incubation is similar to the amount observed in wt nuclei after 8 h of incubation with cisPt. The relatively high concentration of cisPt in the resistant cell nuclei underpins that cisPt resistance in NSCLC is due to a multifaceted mechanism [42].

In conclusion, significant less cisPt was detected in resistant A24 cells than compared to A24-wt cells. It has been shown that SC-ICP-MS can also measure metal drugs in isolated cell organelles, such as cell nuclei. This study demonstrated that metals above a certain detection limit can be measured in subcellular compartments by SC-ICP-MS. However, only one subcellular compartment was studied in these experiments; namely, the nucleus. Other subcellular compartments are of interest, but currently the detection limit of the instrument is a limiting factor. In addition, it was shown that the difference in cisplatin uptake by the cells strongly correlates with different cell volumes. Demonstrated by using lognormal distribution, thus showing that a heterogeneous distribution of cisplatin uptake into cells is unlikely due to some other unknown mechanism. In conventional ICP-MS, this information is lost because it is a mass analysis. Overall, the SC-ICP-MS method allowed a faster quantification of intracellular and nuclear Pt concentrations than classical ICP-MS because pelleted cells or nuclei were resuspended directly in water and PBS without the need of fixation. Moreover, there is no loss of intracellular metal because there is no membrane permeabilization in SC-ICP-MS, which is a common shortcoming in ICP-MS. These findings pave the way for future applications investigating the potency and efficacy of novel metallodrugs developed for precision medicine.

## 4. Materials and Methods

### 4.1. Cell Cultivation

A24, A24dPt2, A24dPt4, and A24dPt8 were included for evaluation of cisPt accumulation in this study [23]. The A24 sublines, were cultured in RPMI 1640 without riboflavin, phenol red, and antibiotics, buffered with 4.5 mM HEPES (BioConcept, Allschwil, Switzerland), supplemented with 10 % (*v/v*) fetal bovine serum (FBS) (Thermo Scientific, Waltham, MA, USA) as the only source of flavins, and 13.5 mM NaHCO_3_. At subcultivation, cell monolayers were rinsed with PBS and exposed for 3 min to StemPro Accutase Cell Dissociation Reagent (Thermo Scientific, Waltham, MA, USA) at 36.5 °C. Detached cells were re-suspended in culture medium. Cell densities were determined using MoxiFlow cytometer according to manufacturer instructions (Orflo Technologies, Ketchum, ID, USA). Experiments and subcultivations were performed using light with wavelengths above 520 nm to prevent photochemical artifacts [43]. Culture vessels and test plates were incubated in the dark at 36.5 °C below 3.5 percent CO_2_ (*v/v*) and humidified air.

### 4.2. Cell Preparation for SC-ICP-MS

One day before starting the experiments, 5.5 × 10^5^ cells/mL were plated in 6 well plates. Then, 30 µM cisPt (Sandoz, Holzkirchen, Germany) was added and incubated for either 5 min, 2, 4, 8 or 16 h under standard conditions. Cells were washed twice with ice-cold PBS (Gibco, NY, USA) and detached with 0.5 mL of StemPro Accutase Cell Dissociation Reagent in order to remove any cisPt adsorbed to the cell wall. Detached cells were resuspended in culture media and cell density, as well as cell volume, was measured using the MoxiFlow cytometer according to the manufacturer’s instructions (Orflo Technologies, Ketchum, ID, USA) [23]. 5.5 × 10^5^ cells were centrifuged for 5 min at 500 g and 4 °C. Supernatant was discarded and the pellet was washed once with PBS. Pellets were kept on ice until measurement.

### 4.3. Nuclei Isolation for SC-ICP-MS Analysis

Nuclei isolation was performed as described previously with minor modifications using Nuclei isolation Kit: Nuclei EZ Prep (Merck KGaA, Darmstadt, Germany) [44]. Briefly, 10^6^ A24 or A24dPt8 cells were washed twice with PBS. The pellet was resuspended in EZ Lysis Buffer, vortexed and incubated on ice for 5 min. Afterwards the sample was centrifuged at 500 g for 5 min at 4 °C. This step was repeated one. The pellet was re-suspended in 500 µL EZ buffer containing 0.25 M sucrose and layered on top of 500 µL EZ buffer containing 0.5 M sucrose. Finally, the nuclei were obtained by centrifugation at 500 g for 10 min at 4 °C and washed once with EZ buffer. Nuclei were re-suspended in PBS. Number of nuclei and nuclei volumes were determined using an automated particles counter (Orflo technologies, Ketchum, ID, USA). Samples were kept on ice.

### 4.4. SC-ICP-MS Measurements

Single cell ICP-MS experiment were performed using Perkin Elmer’s NexION ICP-MS (Perkin Elmer Waltham, MA, USA) which was set up with specific material for single cell experiments as previously described and with minor modifications [45]. The instrumental conditions applied are shown in Table 1. The transport efficiency was calibrated with 50-nm gold nanoparticles (NanoComposix, San Diego, CA, USA) with a concentration of 10^5^ particles/mL. The device was calibrated for platinum using standard solutions prepared in 2% HNO_3_ with 1, 5, 10, and 20 ppb Pt. To generate the calibration curve, averaged intensities for Pt over a measuring time of 60 s were used. Cells or nuclei were re-suspended in PBS and 1:3 diluted in H_2_O resulting in a final concentration of 10^5^ particles/mL. Cells were passed through a 25-gauge needle prior to measurement. Every experiment was performed in triplicates at least. Results were processed using the Syngistix ^TM^ Single Cell Application Software (Perkin Elmer, Waltham, MA, USA).

### 4.5. Western Blotting

Protein purity and absence of cytoplasmic contamination, was assessed with antibodies against lamin A/C (inner nuclear membrane marker), SERCA2 ATPase (endoplasmic reticulum marker), and GAPDH (cytoplasmic marker) using Western blotting as described elsewhere [44]. These antibodies were obtained from Abcam (Abcam, Cambridge, UK). After washing the cells in ice-cold PBS, the cell pellet was resuspended in Triton based RIPA lysis buffer supplemented with the protease inhibitor cocktail, complete Mini, EDTA-free (Merck KGaA, Darmstadt, Germany). Lysates were centrifuged at 8609 rcf and the supernatant was mixed with NuPAGE™ LDS Sample Buffer supplemented with NuPAGE™ Sample Reducing Agent (Thermo Scientific, Waltham, MA, USA). SDS-PAGE was performed using NuPAGE™ 4–12% Bis-Tris gels and NuPAGE™ MOPS SDS running buffer (Thermo Scientific, Waltham, MA, USA). Proteins were transferred using activated porous 0.45 μm polyvinylidene fluoride (PVDF) membranes (Thermo Scientific, Waltham, MA, USA). After blocking the membranes in TBS-T (0.15 M NaCl, 50 mM Tris-HCl, 0.1% Tween 20, pH 7.6) containing 5% milk powder and 1% bovine serum albumin (Merck KGaA, Darmstadt, Germany), a primary antibody was used to detect the protein of interest. After washing the membranes with TBS-T, they were incubated with secondary horse radish-peroxidase conjugated secondary antibodies (Agilent technologies Dako, Santa Clara, CA, USA). Membranes were developed using SuperSignal^®^ West Dura Extended Duration Substrate (Thermo Scientific, Waltham, MA, USA) and visualized using Gel Documentation system Fusion-FX7-820 (Vilber Lourmat Sté, Collégien, France)

### 4.6. Statistics

Multiple unpaired t tests were performed using GraphPad Prism 8.0 Software [46].

## Figures and Tables

**Table 1:** SC-ICP-MS conditions.

Parameter	Value
Sample Uptake Rate	10 µL/min
Nebulizer	N8152496 REV A CytoNeb
Spray chamber	Asperon
Injector	2.0 mm id Quartz
Sample Line	N8152489 REV A
RF Power	1600 W
Neb Gas Flow	0.38–0.42 L/min
Makeup Gas Flow	0.61 L/min
Platinum Isotope	195 amu
Dwell time	50 µs
Sample Analysis Time	100 s

## Data Availability

The data presented in this study are available on request from the corresponding author.
